# Process Intensification of a Napabucasin Manufacturing Method Utilizing Microflow Chemistry

**DOI:** 10.1021/acsomega.2c07997

**Published:** 2023-03-09

**Authors:** Hirotsugu Usutani, Kenji Yamamoto, Kazuki Hashimoto

**Affiliations:** Technology Research & Development Division, Process Research & Development Laboratories, Sumitomo Pharma Co., Ltd., Kasugade-naka 3-1-98, Konohana-ku, Osaka 554-0022, Japan

## Abstract

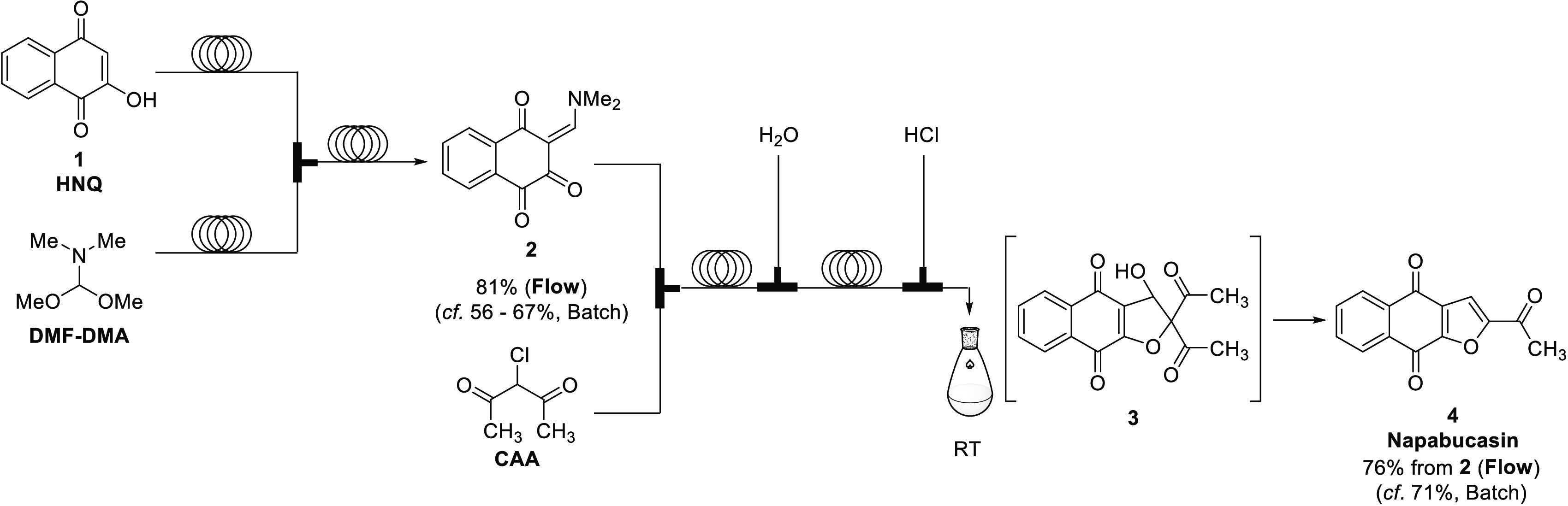

Microflow chemistry is one of the newest and most efficient technologies used today for the safe and effective production of medicines. In this paper, we show the use of this technology in the development of a manufacturing method for napabucasin, which has potential in the treatment of colorectal and pancreatic cancers. In conventional “batch-type” reactor systems, the generation of side products can be controlled with traditional techniques such as reagent reverse-addition and temperature control. However, there is a limitation to which the yield and purity can be improved by these methods, as both are constrained by the efficiency of heat/mass transfer. Applying microflow chemistry technology alters the parameters of the constraint through the use of precise mixing in a microchannel, which offers increased possibility for improving yields and process intensification of the napabucasin process. Reported herein is a proof-of-concept study for the scale-up production of napabucasin using microflow chemistry techniques for manufacturing at the kilogram scale.

## Introduction

For more than a century, active pharmaceutical ingredient (API) production has been executed in the batch mode in traditional manufacturing plants, but an increasing number of API processes have been developed over the last decade or so where flow-mode operations have been used. In the field of scale-up process research, much effort has been made to improving API processes, and flow mode has been used for process intensification.^1^ Comparison of flow and batch process development shows that there are fewer scale-up factors involved in flow process development,^2^ and examples of scale-up using flow chemistry are becoming increasingly common.^3^

Flow chemistry efficiently utilizes reaction volume in the range of microliters to liters, and though the reactor space is compact compared to traditional manufacturing facilities, the productivity is not inferior to conventional batch reactors.^4^ Furthermore, flow mode may offer superior features for safety, efficiency, and cost competitiveness in the production stage.^5^ The product “harvest” obtained from a microflow chemistry process is derived from “scaling effects”, which have been well investigated by Mae and co-workers.^6,7^ They reported that viscous force, surface tension, and heat transfer are correlated with spatial distance, and molecular diffusion becomes dominant in microspaces.^8^ The general flow regime in a microreactor is classified as laminar flow, and intensive vortices can be generated by junction and bending geometries. The microflow mode with appropriate flow velocity achieves a much faster mixing than in the batch mode.^9,10^

The development of effective mixing conditions in the batch mode requires experimenting with different aspects of the manufacturing process, which includes the stirring components as well as the reactor design.^11^ Mixing effects are often discussed in process development, and improved, more practical mixers have been developed in recent years.^12^ In Mae et al.’s paper on scaling effects,^6^ the order of magnitude difference for mixing in vessels of different sizes is described. In contrast, mixing in the flow mode is usually investigated by changing the channel size and flow rate. With the appropriate choice of these parameters, both fast mixing and high-throughput synthesis can be achieved with micro- to milli-scale reactors.^13^

In this paper, process intensification of napabucasin, utilizing the benefits of mixing in a microflow system, will be reported. Furthermore, we disclose feasibility studies on the kg-scale manufacturing of an intermediate and the scale-up production of napabucasin in a semiflow mode.

## Results and Discussion

### Development of the Napabucasin Process (Batch Mode)

Napabucasin^14^ has been developed as a drug candidate for the treatment of pancreatic cancer, but a phase III study investigating the efficacy of napabucasin when given with the standard-of-care chemotherapy to patients with metastatic pancreatic adenocarcinoma (mPDAC) was discontinued.^15,16^

**Scheme 1:**
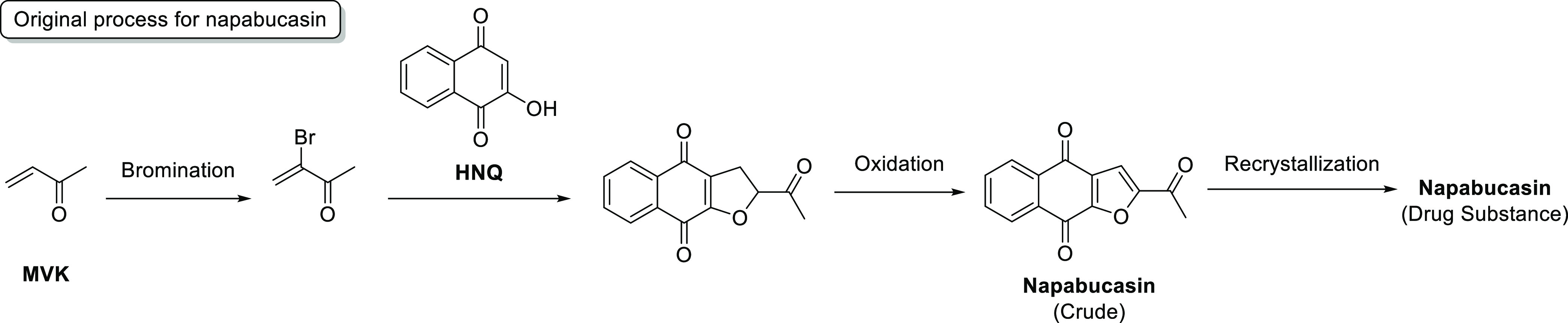


The original process for napabucasin, developed for medicinal research, is shown in Scheme 1; however, the process required the use of methyl vinyl ketone (MVK) as a key starting material. As is well known, MVK is a toxic compound; hence, further process improvements are desired.

**Scheme 2:**
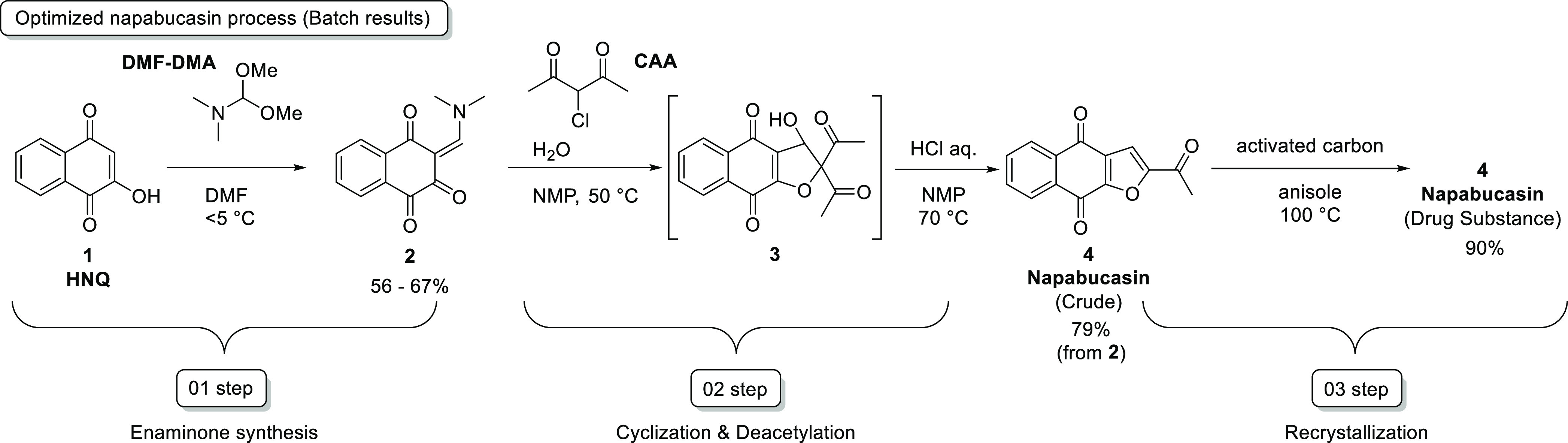


Much effort was spent on the development of a batch process for napabucasin, and a batch-style validated commercial process was developed in our laboratories.^17^ One of the processes adopted 2-hydroxy-1,4-naphthoquinone (HNQ, **1**) as a regulatory starting material and *N*,*N*-dimethylformamide dimethyl acetal (DMF–DMA) was selected as a reagent to form the enaminone **2**. This species was then treated with 3-chloro-2,4-pentanedione (chloro-acetylacetone, CAA) to afford the hydroxy-hydrofuran **3**, followed by submission of that to conditions promoting elimination of acetic acid to produce napabucasin (**4**), with subsequent purification and recrystallization (Scheme 2).

### Development of Enaminone **2** Process Utilizing a Flow Mode

**Figure 1:**
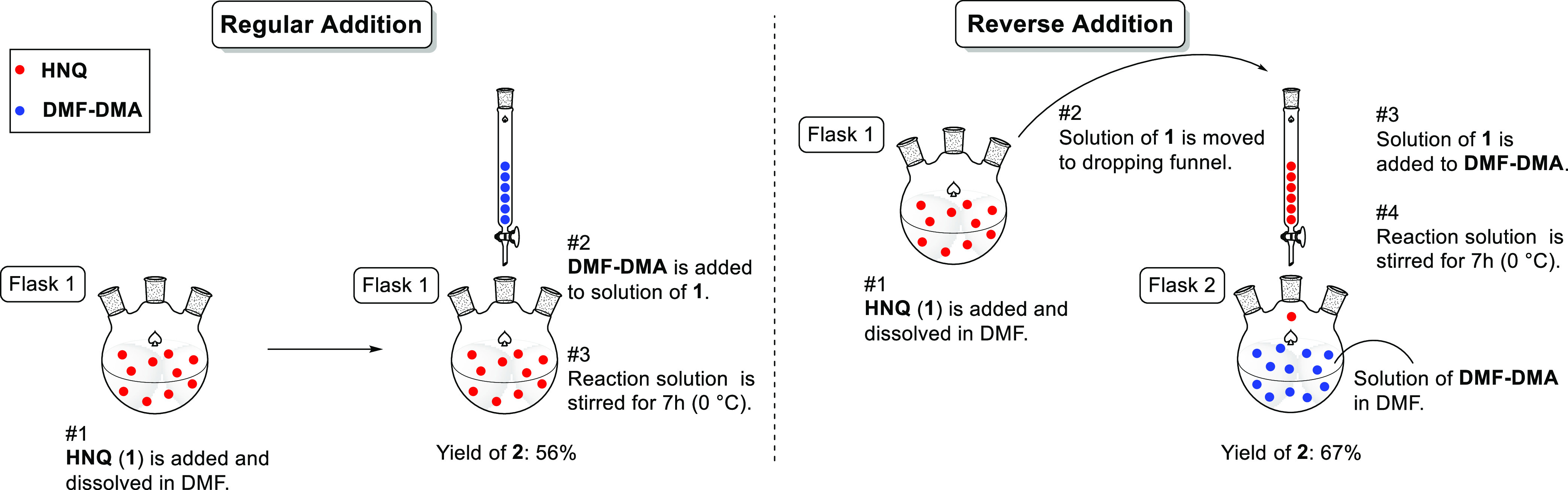
Regular and reverse addition methods for synthesizing **2**.

**Scheme 3:**
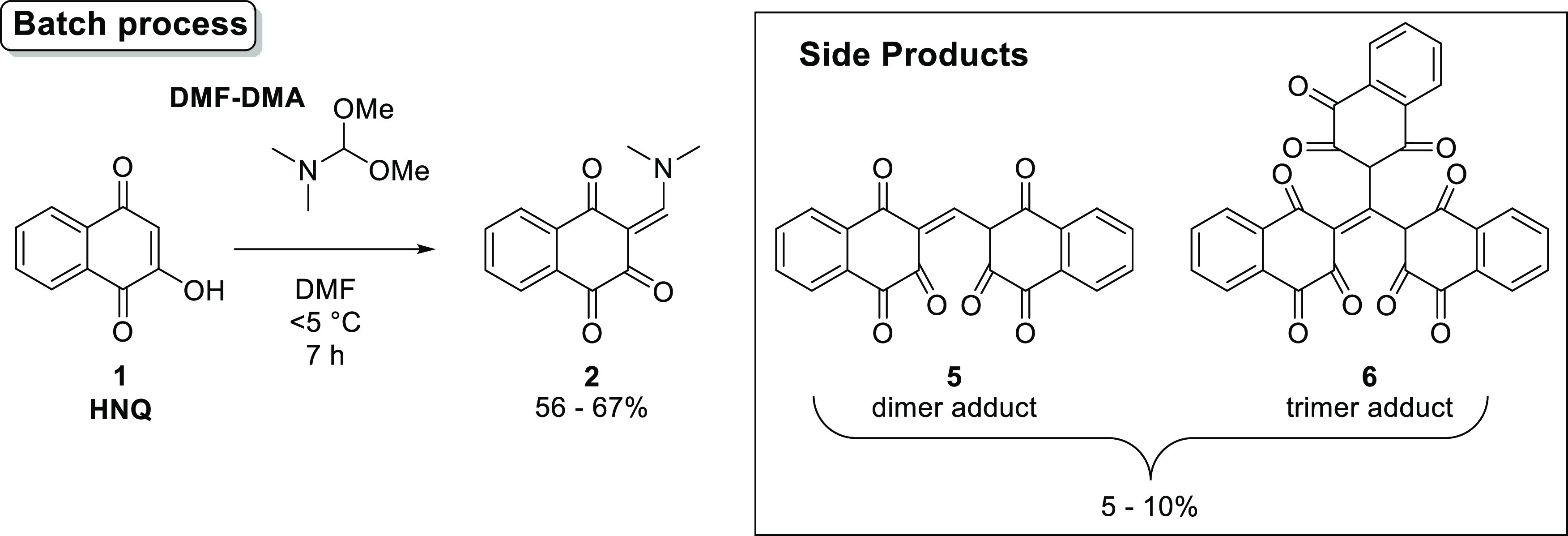


Although many reagents and conditions were investigated for the batch process of napabucasin, the yields for the condensation of HNQ (**1**) and DMF–DMA (01 step) were only moderate. Some side reactions were found to be unavoidable, and the generation of side products resulted in only moderate yield for the 01 step, even after optimization (Scheme 3). The side products were the result of over-reactions to give the dimer (**5**) and trimer (**6**) adducts through the reactions of **1** and **2**, and **1** and **5**. Therefore, a reverse addition method was employed, in which a solution of **1** in DMF was added to a DMF–DMA solution (Figure 1).

**Figure 2:**
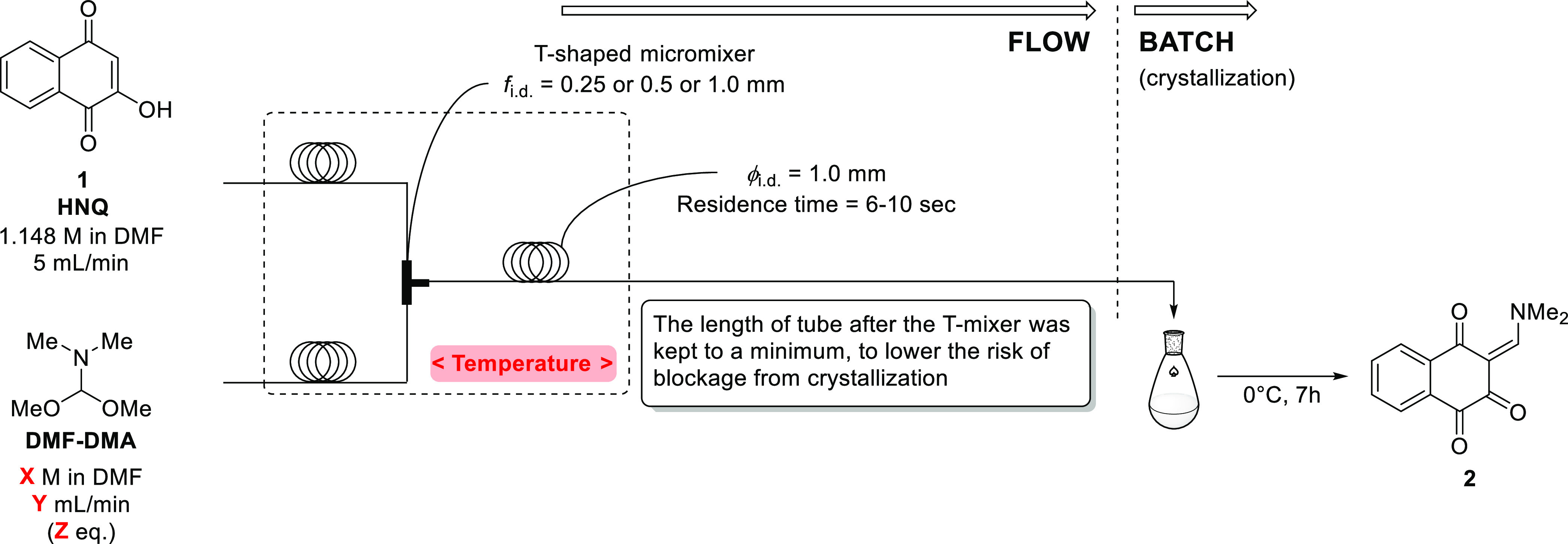
Setup for a microflow feasibility study of enaminone **2** synthesis.

When a reverse addition method was used for the reaction, the yield of the target compound (**2**) was slightly increased but generation of the side products was still evident, with ca. 5–10% of the dimer or trimer adducts being formed. Thus, it was thought that this step might benefit from the improved mixing efficiency of a microflow approach to minimize generation of the dimer and trimer adducts,^18,19^ and a microflow reactor setup was constructed for a feasibility study (Figure 2 and Table 1).

**Table 1:** Feasibility Study of Enaminone **2** Synthesis by the Microflow Mode

entry	micromixer (ϕ_i.d._, mm)	*X* (M)	*Y* (mL/min)	*Z* (equiv)	temperature (°C)	isolated yield^a^ (%)
1	0.5	1.723	5	1.5	0	69
2	1.0	1.723	5	1.5	0	62
3	0.5	neat	1.13	1.5	0	70
4	0.25	neat	1.13	1.5	0	76
5	0.25	neat	1.13	1.5	–10	76
6	0.25	neat	1.13	1.5	–20	73
7	0.25	neat	1.51	2.0	–10	81^b^
8	0.5	neat	1.13	1.5	0	67^c^

a A minimal length of tube was used after the T-mixer to reduce the risk of blockage.

b Less than 0.1% dimer adduct **5** was generated, which was completely removed by filtration, and no trimer adduct **6** was observed.

c V-shaped mixer was used for mixing.

First, a T-shaped micromixer was used for mixing a solution of **1** (HNQ) in DMF and a solution of DMF–DMA in DMF. The microflow reactor system was cooled in an ice bath, and the reaction mixture was collected and stirred for 7 h in a flask for crystallization. However, the desired product (**2**) was only obtained in about the same yield as for the batch mode (entry 1), and when a wider diameter micromixer was used, presumably with a lower mixing efficiency, the yield decreased (entry 2). Subsequently, to simplify the microflow process for further studies, neat DMF–DMA was employed, and it was found that there were no disadvantages to using the neat reagent (entry 3). Then, on comparing entries 1 and 2 or entries 3 and 4, it can be seen that the reaction yield improved as the T-micromixer internal diameter was decreased. Thus, it is clear that the mixing efficiency is increased for the smaller-sized micromixers, which is one of the advantageous scaling effects of microflow.^20^

Furthermore, the effects of the mixing temperature and the equivalent of DMF–DMA were investigated (entries 5, 6, and 7), and the optimum condition for the reaction was found at −10 °C using 2 equiv (entry 7). Notably, under those conditions, the amount of the dimer adduct was reduced and the trimer adduct was eliminated altogether.

As for the micromixer design, we also investigated the use of a V-shaped mixer (entry 8), but no improvement in yield was obtained, so the most simple and widely available T-shaped mixers were used for all subsequent experiments.^21^

### Scale-Up Production of Enaminone **2** Utilizing Flow Technology

**Figure 3:**
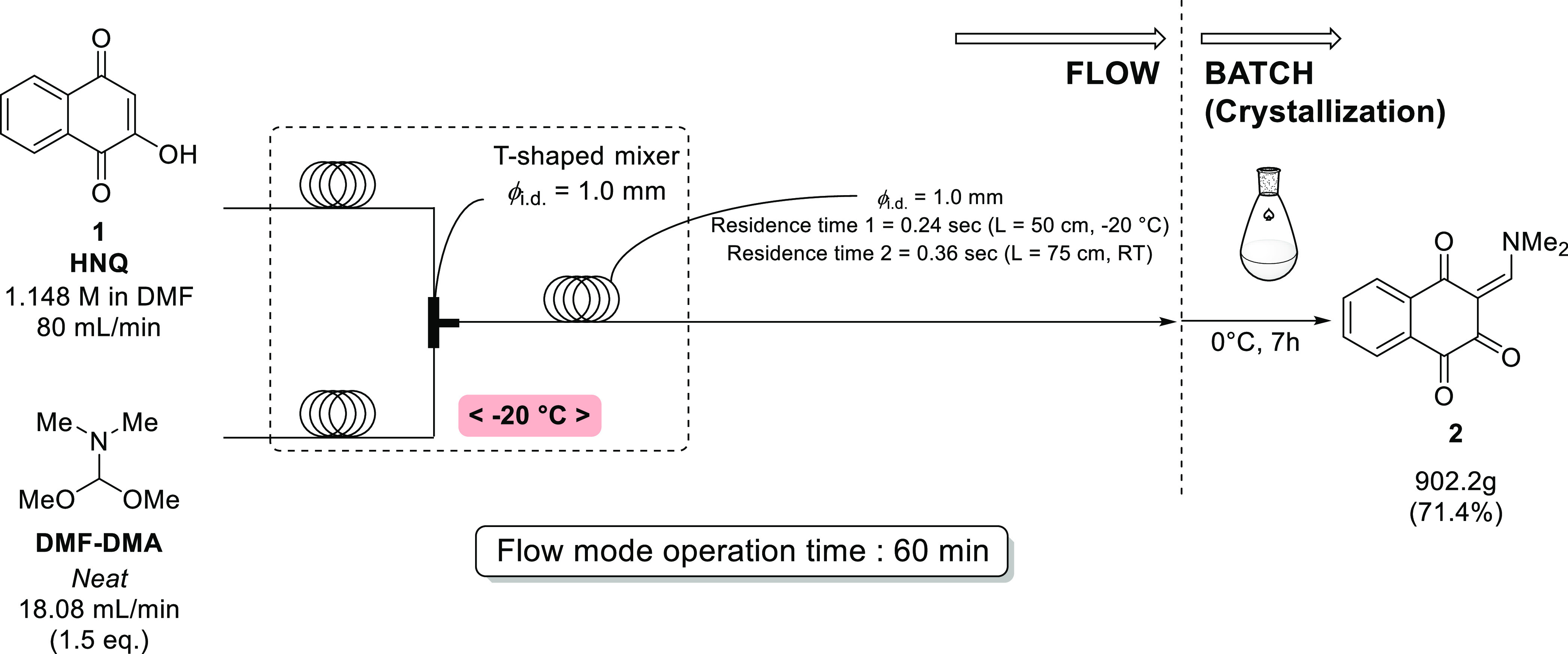
Scale-up production for **2**.

A scale-up production for enaminone **2** was investigated, with the flow rates increased from the initial lab conditions (HNQ: 5 mL/min; DMF–DMA: 1.13 mL/min) to improve productivity (HNQ: 80 mL/min; DMF–DMA: 18.08 mL/min). Due to a concern about the efficiency of thermal exchange in the 1.0 mm-diameter reactor at higher flow rates, the reaction bath temperature was lowered to −20 °C. Also, the number of equivalents of DMF–DMA was lowered to 1.5 equiv, in anticipation that the mixing efficiency would be somewhat greater at higher flow rates due to turbulent mixing adding with diffusion mixing, which would allow the reagent to be used more economically. The result showed that over 900 g of **2** could be manufactured in a reasonable yield, with an operation time for the flow mode of 1 h (Figure 3). The product **2** was of very high quality, without any detectable dimer or trimer side product adducts, and it could be used directly in the subsequent reaction.

By increasing the inside diameter of the micromixer to 1.0 mm for scale-up, it was thought that the risk of clogging would be reduced. It was also anticipated that any decrease in the mixing efficiency due to the widening of the inner diameter would be compensated for by increasing the flow rate 16 times.^22^ Although the isolated yield of 71% was somewhat lower than desired, it was noted that some cavitation occurred in the pumps during operation, which would have caused imprecise flow rates and decreased yields. It was estimated that cavitation prevented ca. 10% of the **1** (HNQ) solution from correctly entering the flow system. It is thought that the cavitation issues might be solved by widening the suction tube to the pump and/or taking advantage of back-pressure regulators soon after and/or just before the micromixer (before the crystallization starts). In addition, the actual temperature and inside diameter of the T-shaped mixer may be slightly different from the optimal condition shown in Table 1. It is thought that these multiple factors might be the reasons for the yield decrease. However, the ease of flow-mode scale-up was evident, as even the manufacturing could be carried out at the “lab-scale” with a 5 L flask.

### Flow Chemistry Process for Napabucasin (**4**) Synthesis

As described above, flow chemistry was applied to enaminone synthesis, and the advantage of a microflow process, especially the efficiency of mixing in the flow mode, was demonstrated. Next, development of a semiflow chemistry process for napabucasin (**4**) was carried out.

**Figure 4:**
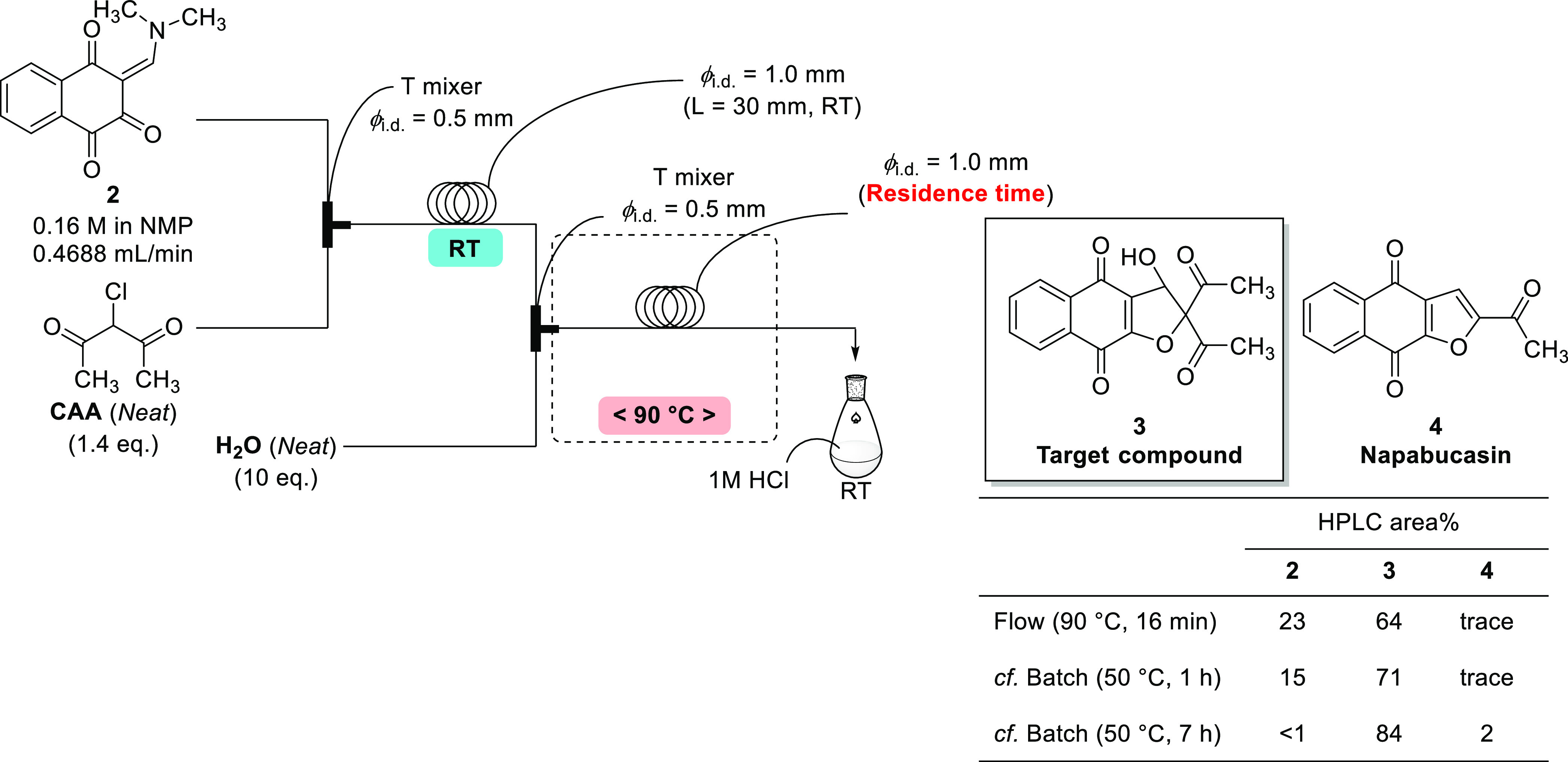
Feasibility study of flow synthesis for **3**.

The batch process for napabucasin consists of two steps, and it is necessary to consider each step separately. First, a feasibility study of the cyclization step was performed and generation of the target compound (**3**) was confirmed (Figure 4).

**Figure 5:**
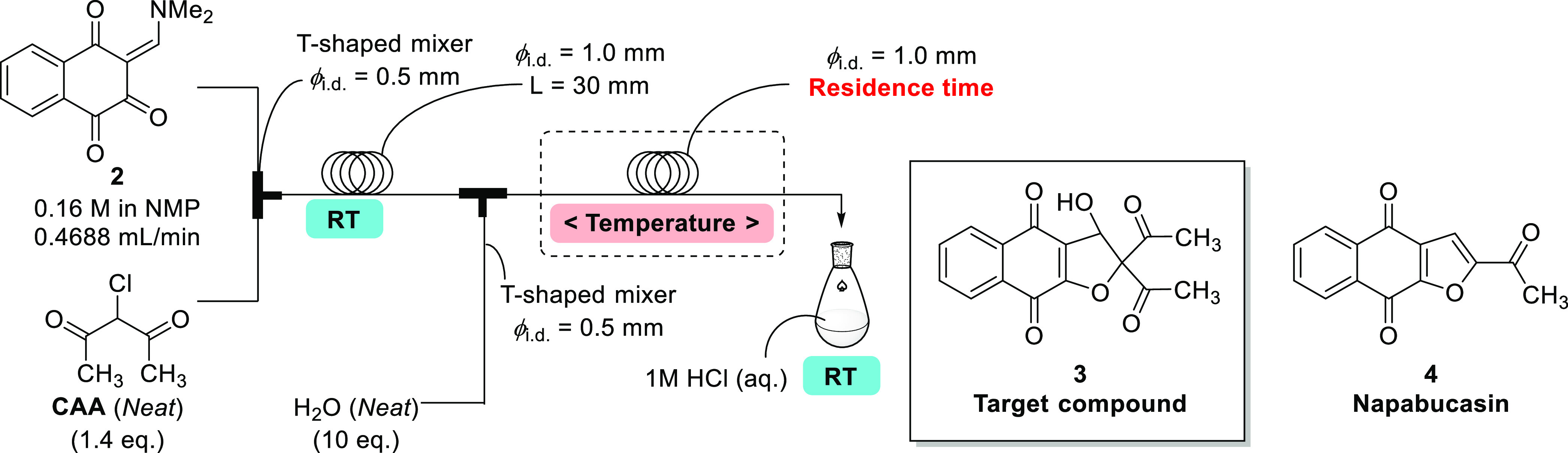
Optimization of flow synthesis for **3**.

When the cyclization temperature was raised to 70 °C or more in a batch, the reaction solution became a slurry and many side products were generated. However, in flow, we could successfully accelerate the cyclization reaction using a higher reaction temperature (90 °C) than for the optimum batch temperature of 50 °C. With heating in the flow mode, even though the residence time was only 16 min, the reaction profile was almost equal to that obtained after 1 h in the batch mode (the reaction required more than 7 h to complete in batch). Therefore, the cyclization step in flow was optimized further by investigating the effect of temperature and residence time (Figure 5 and Table 2).

**Table 2:** Optimization of the Intermediate **3** Synthesis by Flow

			yield^a^		
entry	temperature (°C)	residence time (min)	**2**	**3**	**4**
1	90	16	23	64	trace
2	110	16	12	69	2
3^b^	130	16	8	64	7
4	90	32	11	73	1
5	110	32	4	71	3
6	90	64	4	79	1
7^c^	90	62	trace	80	1

a HPLC area%.

b The reaction gave a complex mixture.

c 2.8 equiv of CAA was used.

It was observed that as the temperature was increased, more enaminone intermediate **2** was consumed, and more target compound **3** was generated (entries 1, 2, and 3). However, higher temperatures did not necessarily improve the reaction yield as it appeared that higher temperatures promoted side reactions (entry 3). On increasing the duration of the residence time, the reaction yield was improved (entries 4, 5, and 6). Thus, based on these results, it was thought that a prolonged residence time, without further increase in the reaction temperature, would be optimal. Hence, 1 h residence was used (entry 6), and compound **3** was obtained in good yield (79%). It was also found that a greater excess of CAA enhanced the yield (entry 7), and continuous operation under the conditions of entry 7 for 100 min afforded 1.26 g of napabucasin (70%, isolated yield).

### Proof-of-Concept for Napabucasin Manufacturing by a Semiflow Process

**Figure 6:**
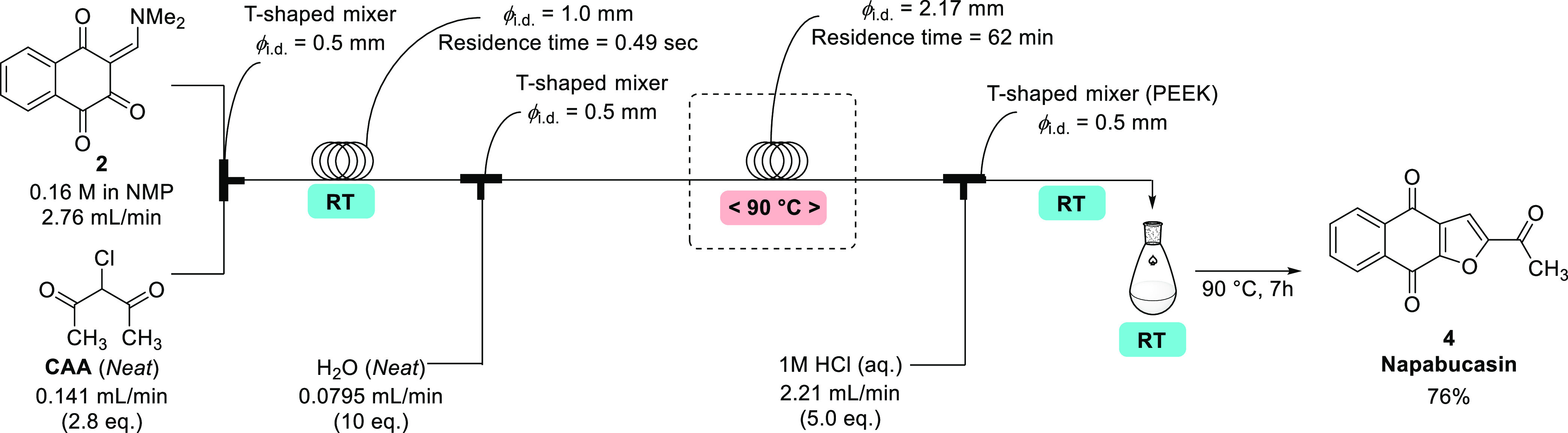
Proof-of-concept manufacturing for napabucasin synthesis by a semiflow process.

Using the conditions of entry 7 in Table 2, a scale-up production for napabucasin synthesis was conducted with ca. 6 times higher flow rates (Figure 6).

**Scheme 4:**
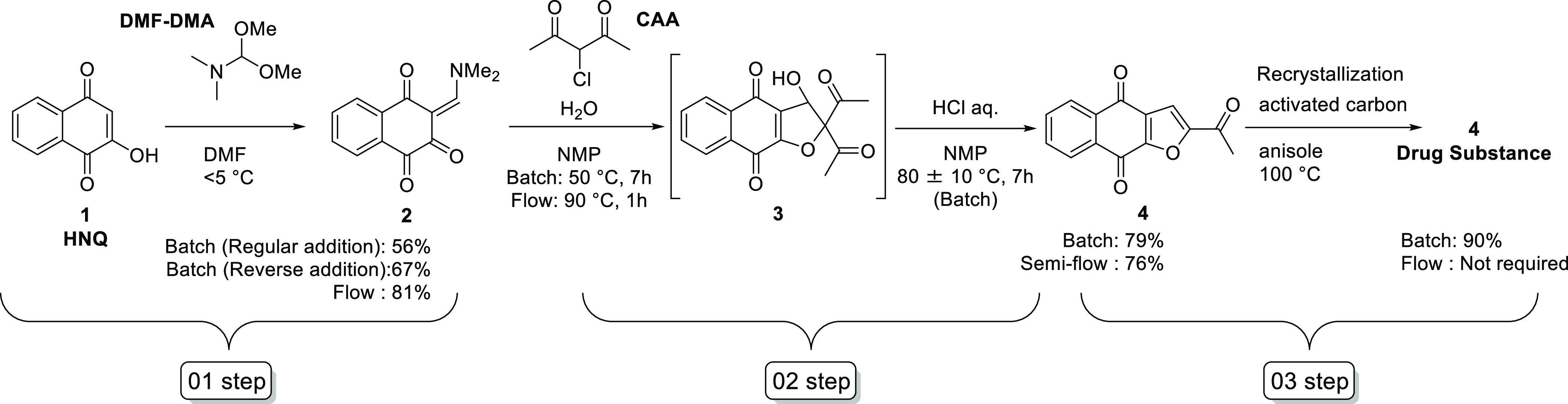


To have sufficient residence time for the cyclization step, a wider inside diameter tube (ϕ_i.d._ = 2.17 mm) was utilized in the flow-heating system. After 9 h operation in the flow mode and the accompanying post-processing in batch (heated to 90 °C for 7 h, then filtrated and dried in a vacuum oven), 43.42 g (76%) of napabucasin was obtained. Notably, the isolated napabucasin showed extremely high purity (99.92%), and subsequent recrystallization was unnecessary (Scheme 4 and Table 3). It is also noteworthy that the total isolated yield from the semiflow system was 62%, which was significantly better than the 40–48% obtained in the batch mode.

**Table 3:** Summary and Comparison of the Process for Napabucasin (Batch vs Flow)

	batch^a^		flow^b^	
step no.	isolated yield (%)	purity^c^ (%)	isolated yield (%)	purity^c^ (%)
01	56^d^–67^e^	99.63	81	>99
02	79	99.75	76	99.92
03^f^	90	99.82	N/A	N/A
total	40–48	N/A	62	N/A

a Maximum 30 kg scale.

b kg scale for the 01 step and multi-g scale for the 02 step.

c HPLC area%.

d Regular addition method.

e Reverse addition method.

f Napabucasin obtained after the 02 step by flow was highly pure, eliminating the need for the 03 step.

### Evaluation of Process Mass Intensity (PMI)

Finally, the process mass intensity (PMI) values were evaluated for the batch and flow processes (Table 4). Surprisingly, the PMI for the 01 step decreased by almost a half for the flow process. With regard to the 02 step, due to the low solubility of **2**, **3**, and **4**, the total quantity of solvent used was increased in flow; however, the total PMI was still improved. The 02 step in batch is a slurry reaction; thus, the volume of the solvent used is less than for the flow process, which requires complete dissolution of the materials.

**Table 4:** Raw Materials Required to Obtain 1 kg of Napabucasin (PMI Evaluation) for Batch and Flow Processes

step no.	items	batch charged amount (kg)	flow charged amount (kg)
01 step	HNQ	1.53	1.18
	DMF–DMA	1.57	1.59
	DMF	11.44	4.46
	MeOH	6.10	4.73
02 step	CAA	1.12	2.09
	H_2_O	3.80	28.47
	conc. HCl	1.09	1.00
	NMP	8.19	35.35
	MeOH	21.84	N/A
03 step	activated carbon	0.05	N/A
	anisole	21.74	
	EtOAc	8.70	
total (PMI)		87.16	78.88

Even though the solvent used in the 02 step was increased, it is noteworthy that we could eliminate the 03 step and reduce the total PMI using a flow process, which further shows how flow processes can contribute to process intensification.

## Experimental Section

### HPLC Method for Step 01

The HPLC method used for IPC analysis as well as for analyzing the purity of 2 employed an Inertsil Diol 5 μm, 4.6 × 150 mm^2^ column maintained at 30 °C. A solution of *n*-hexane/THF/acetonitrile/trifluoroacetic acid (TFA) = 900/200/100/1 was used as the mobile phase, and the analysis was carried out under isocratic conditions. The flow rate was set to 1.0 mL/min, the injection volume was 5.0 μL, and detection was carried out at 250 nm. The total method analysis time was 50 min.

Compounds **1**, **2**, **5**, and **6** eluted at relative retention times (RRTs) of 4.5 min (**1**), 22.5 min (**2**), 13.9 min (**5**) and 26.2 min (**6**).

### General Lab-Scale Experimental Procedure (Flow Synthesis of **2**, Table 1)

All chemicals were purchased from FUJIFILM Wako Pure Chemical Corporation and used without further purification. All solvents used were of reagent grade unless otherwise specified. Two microsyringe pumps (ISIS Ltd., Osaka Japan, Fusion 100) were used to pump the solutions of the two reagents. Feedstock A consisted of compound **1** (HNQ) dissolved in DMF as a 1.148 M solution. Feedstock B was commercially available DMF–DMA (neat) used directly from the supplied bottle. Both feedstocks were maintained under an atmosphere of nitrogen and made up using anhydrous DMF. The reactors were fabricated from stainless steel (SS) tubing with an internal diameter (ID) of 1 mm and an appropriate length defined by the desired residence time (τ) and flow rates. The residence time from the micromixer to the outlet was adjusted by utilizing a suitable tube length determined by the total flow rate of feedstocks A and B (in entry 7, the tube length was 1250 mm and the total flow rate was 6.51 mL/min; therefore, the residence time was 9.0 s). Shimadzu-GLC tee piece (ID 0.50 mm, part number: 6010-72357)^23^ was used as a mixer for feedstocks A and B.

To make sure reactants were sufficiently cooled before mixing, precooling loops (*L* = 50 cm, ID = 1.0 mm) were used, and the reactors for mixing were submerged into a cooling bath set at −10 °C before starting the two pumps. Once a steady flow was attained without blockage (typically after 30 s of continuous operation), the product stream was diverted to a 100 mL flask and collected for 4 min and then stirred for 7 h in an ice bath. The slurry solution was filtrated and washed with MeOH (10 mL, 2 times), followed by drying in a vacuum oven (50 °C) to give 4.26 g of enaminone **2**.

### HPLC Method for Steps 02 and 03

The HPLC method used for IPC analysis as well as for analyzing the purity of 4 employed a Phenomenex Luna 5 μm C18(2) 100 Å, 4.6 × 250 mm^2^ column maintained at 30 °C. A solution of distilled water/methanesulfonic acid = 1000/2 was used as mobile phase A and a solution of acetonitrile/2-propanol/methanesulfonic acid as mobile phase B. The total flow rate was set to 1.0 mL/min, the injection volume was 10 μL, and the detection was carried out at 250 nm. The total method analysis time was 55 min. A gradient was used starting at 25% of mobile phase B, moving to 45% over 20 min, and moving to 85% over 20 min, eluting at 85% B for 5 min and then moving back to 25% over 0.01 min. The final composition was maintained for 10 min at 25% to re-equilibrate the column.

Compounds **2**, **3**, and **4** eluted at relative retention times (RRTs) of 6.7–8.7 min (**2**, broad), 16.1 min (**3**), and 22.6 min (**4**).

### General Lab-Scale Experimental Procedure (Flow Synthesis of **3**, Table 2)

All chemicals were purchased from FUJIFILM Wako Pure Chemical Corporation and used without further purification. All solvents used were of reagent grade unless otherwise specified. Three microsyringe pumps (ISIS Ltd., Osaka, Japan, Fusion 100) were used to pump the solutions of the two reagents. Feedstock A consisted of compound **2** dissolved in NMP as a 0.16 M solution. Feedstock B was commercially available CAA (neat) used directly from the supplied bottle. Feedstock C was distilled water used directly from the supplied bottle. The reactors were fabricated from Teflon (PFA) tubing with an internal diameter (ID) of 1 mm and an appropriate length defined by the desired residence time (τ) and flow rates. The residence time from the second micromixer to the outlet was adjusted by utilizing a suitable tube length determined by the total flow rate of feedstocks A, B, and C (in entry 6 of Table 2, the tube length was 40 m and the total flow rate was 0.5063 mL/min (A: 0.4688 mL/min; B: 0.024 mL/min; C: 0.0135 mL/min); therefore, the residence time was 62 min). A Shimadzu-GLC tee piece (ID 0.50 mm, part number: 6010-72323) was used as a mixer for feedstocks A, B, and C.

The reactors, mixers, and tubing were submerged into a heating bath set at 90 °C (entry 6) before starting the three pumps. Once a steady flow was attained, the product stream was diverted to a 100 mL flask, quenched by HCl, and subsequently analyzed by HPLC.

### Scale-Up Production

#### Flow Chemistry Process for Enaminone 2 (Scale-Up Synthesis of **2**, Figure 3)

Two plunger pumps (Intelligent Pump UI-22-410P, FLOM Corporation) were used to pump the solutions of the two reagents. Feedstock A consisted of compound **1** dissolved in anhydrous DMF as a 1.148 M solution, and it was pumped at 80.0 mL/min. Feedstock B was commercially available DMF–DMA (neat) used directly from the supplied bottle and pumped at 18.08 mL/min. All feedstocks were maintained under an atmosphere of nitrogen. The reactors were fabricated from SS tubing with an ID of 1.0 mm and 1250 mm length. The residence time from the micromixer to the outlet was 0.6 s. Shimadzu-GLC tee pieces (ID 1.0 mm, custom-made item) were used as mixers. The precooling loops (*L* = 0.5 m, ID = 1.0 mm) and reactors for the mixing step were submerged into a cooling bath set at −20 °C before starting the two pumps. After a steady flow was attained, the product stream was collected in a 5 L flask for a total of 60 min, before the collected product was stirred for 7 h in an ice bath. The slurry solution was filtrated and washed with MeOH (2.4 L, 2 times) and then dried in a vacuum oven (50 °C) to give 902.2 g of enaminone **2**.

#### Proof-of-Concept for Napabucasin Manufacturing Using a Flow Process (Scale-Up Synthesis of **4**, Figure 6)

Two plunger pumps (Intelligent Pump UI-22-410P) were used to pump the solutions of the two reagents (compound **2** and HCl), and two syringes were used to flow CAA (neat) and H_2_O (neat).

Feedstock A consisted of compound **2** dissolved in NMP as a 0.16 M solution and was pumped at 2.76 mL/min. Feedstock B was commercially available CAA (neat) used directly from the supplied bottle and pumped at 0.141 mL/min by a microsyringe pump (ISIS Ltd., Osaka, Japan, Fusion 100). Feedstock C was distilled water (neat) used directly from the supplied bottle and pumped at 0.0795 mL/min by a microsyringe pump (ISIS Ltd., Osaka, Japan, Fusion 100). Feedstock D consisted of a 1 M HCl aqueous solution and was pumped at 2.207 mL/min. Although feedstocks A and D were maintained under an atmosphere of nitrogen, feedstocks B and C were not.

The reactors were fabricated from a PFA tubing with an ID of 1.00 mm and an appropriate length defined by the desired residence time (τ) and flow rates (tube for the ring closure reaction was ID of 2.17 mm, SUS). The residence time for the mixing of **2** and CAA was set to 0.49 s, and after the mixing with H_2_O, the residence time was set to 62 min (only here, the tube ID was 2.17 mm). The residence time from the mixing point of the HCl addition to the outlet was set to 9.1 s.

Shimadzu-GLC tee pieces (SS, ID 0.50 mm, part number: 6010-72327) were used as mixers for feedstocks A, B, and C. For mixing feedstock D, Shimadzu-GLC T-pieces (PEEK, ID 0.50 mm, part number: 6010-72323) were used as mixers.

Although all reagents were mixed at room temperature, the tube reactor for ring closure (*L* = 50 m, ID = 2.17 mm) was submerged into an oil bath set at 90 °C before starting the three pumps. After a steady flow was attained, the product stream was collected and the process was run for a total of 9 h. The solution was heated to 90 °C for 7 h to afford 43.42 g of napabucasin in 99.92% HPLC purity.

## Conclusions

Process development and intensification of napabucasin synthesis using a flow method was performed, and a clear advantage of microflow chemistry for avoiding over-reactions was found. Thus, formation of the dimer and trimer adducts that are usually generated in the batch mode was eliminated in the flow process due to the rapid and efficient mixing that occurs in micromixers.

In batch synthesis, reverse addition methods are frequently used to reduce over-reactions, and they are one of the general methods to intensify processes. However, it is clear that the mixing efficiency of a batch mode reaction never surpasses that of a microflow system. Thus, when a reverse addition in batch mode is being considered, it is appropriate to also investigate the application of microflow technology. Mixing in microspace will mix substances precisely and reduce side reactions, which will then contribute to process intensification. Also, it is noteworthy that the application of flow technology to drug substance synthesis can contribute to improving the quality of APIs by the elimination of impurities arising from side reactions. Furthermore, we showed how a flow process can improve the PMI.

As described in this paper, a flow chemistry process can mitigate risks in scale-up production. Although it is difficult to make all processes continuous,^24^ we expect that flow synthesis technology will be used together with conventional batch technology to create synergies, and the number of processes that include flow chemistry will continue to increase.

## Supporting Information Available

Detailed data for HPLC data (PDF)
