# Supplementary material for: Process Intensification of a Napabucasin Manufacturing Method Utilizing Microflow Chemistry
Source: ACS Omega. 2023 Mar 9;8(11):10373–82. doi: 10.1021/acsomega.2c07997 (PMC10034843; doi:10.1021/acsomega.2c07997)
Supplement: Supplementary file 1 — ao2c07997_si_001.pdf [file ao2c07997_si_001.pdf]

Supporting Information

Process Intensification of a Napabucasin Manufacturing Method Utilizing Microflow Chemistry

Hirotsugu Usutani[1*], Kenji Yamamoto[1], and Kazuki Hashimoto[1]

*Email: hirotsugu.usutani@sumitomo-pharma.co.jp

[1] Sumitomo Pharma Co., Ltd., Technology Research & Development Division, Process Research & Development Laboratories, Kasugade-naka 3-1-98, Konohana-ku, Osaka, 554-0022, Japan

HPLC Method for step 01.

The HPLC method used for IPC analysis as well as for analyzing the purity of enaminone (**2**) employed an Inertsil Diol 5um, 4.6×150 mm column maintained at 30 ˚C. A solution of n-Hexane / THF / Acetonitrile / Trifluoroacetic acid (TFA) = 900 / 200 / 100 / 1 was used as Mobile Phase and the analysis was carried out at Isocratic condition. The flow rate was set to 1.0 mL/ min, the injection volume was 5.0 μL and detection was carried out at 250 nm. The total method analysis time was 50 min. Compounds 1, 2, 5, and 6 eluted at a relative retention time (RRT) of 4.5 min (**1**), 22.5 min (**2**), 13.9 min (**5**), 26.2 min (**6**).

Figure S1. HPLC analysis of compound **2** (Table 1, entry 7)

<Chromatogram>

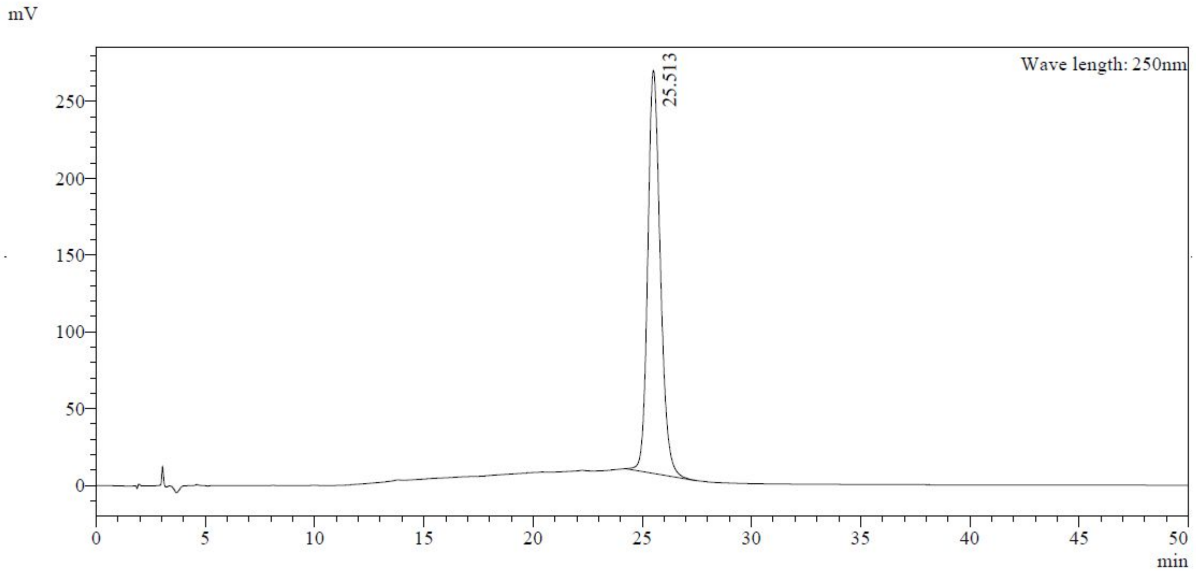

<PeakTable>

Wave length: 250nm

Peak#	Ret. Time	Area	Height	Conc.	Unit	Mark	Name
1	25.513	10526633	262435	100.000		M	
Total		10526633	262435				

Figure S2. HPLC analysis of filtrate in enaminone (**2**) synthesis (Table 1, entry 7)

<Chromatogram>

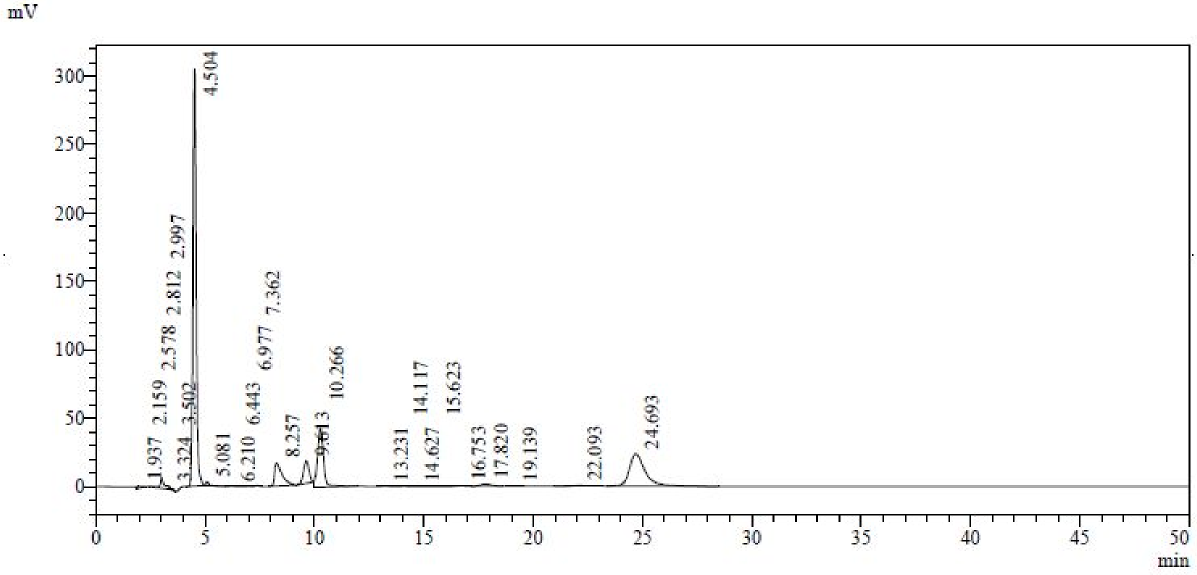

<PeakTable>

Wave lengh: 250nm

Peak#	Ret. Time	Area	Height	Conc.	Unit	Mark	Name
1	1.937	14815	1788	0.243			
2	2.159	3272	473	0.054		V	
3	2.578	4871	379	0.080			
4	2.812	8262	936	0.135		V	
5	2.997	93404	8118	1.529		SV	
6	3.324	3582	524	0.059		T	
7	3.502	6022	972	0.099			
8	4.504	3132802	304541	51.286		M	HNQ (compound 1)
9	5.081	26279	2413	0.430			
10	6.210	2875	253	0.047			
11	6.443	1983	136	0.032		V	
12	6.977	2382	255	0.039			
13	7.362	8097	623	0.133			
14	8.257	405643	16881	6.641			DMF
15	9.613	279367	16060	4.573			aldehyde form of compound 2
16	10.266	841730	43067	13.780			
17	13.231	6405	306	0.105			
18	14.117	5157	256	0.084			
19	14.627	1957	100	0.032		V	dimer (compound 5)
20	15.623	3376	112	0.055			
21	16.753	13030	327	0.213			
22	17.820	55381	1611	0.907		V	
23	19.139	1943	76	0.032			
24	22.093	9000	191	0.147			
25	24.693	1176826	23595	19.266			compound 2
Total		6108460	423995				

Figure S3. HPLC analysis of filtrate in enaminone (**2**) by batch

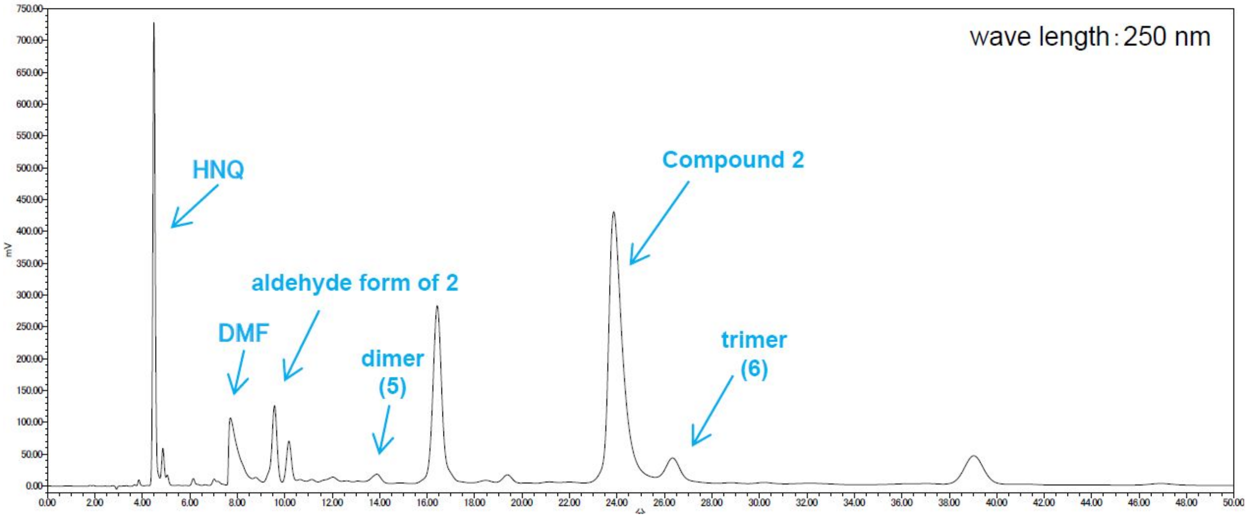

HPLC Method for Step 02 & 03.

The HPLC method used for IPC analysis as well as for analyzing the purity of 4 employed a Phenomenex Luna 5um C18(2) 100Å, 4.6×250 mm column maintained at 30 ° C. A solution of Distilled water/Metanesulfonic acid = 1000/2 was used as Mobile Phase A and a solution of Acetonitrile/2-Propanol/ Metanesulfonic as Mobile Phase B. The total flow rate was set to 1.0 mL/ min, the injection volume was 10 μL and the detection was carried out at 250 nm. The total method analysis time was 55 min. A gradient was used starting at 25 % of Mobile Phase B, moving to 45 % over 20 min, and moving to 85% over 20min eluting at 85 % B for 5 min and then moving back to 25 % over 0.01 min. The final composition was maintained for 10 min at 25 % to re-equilibrate the column.

Compounds 2, 3, and 4 eluted at a relative retention time (RRT) of 6.7-8.7 min (**2**, broad), 16.1 min (**3**), 22.6 min (**4**).

Figure S4. HPLC analysis of intermediate **3** synthesis by flow (Table 2, entry 6)

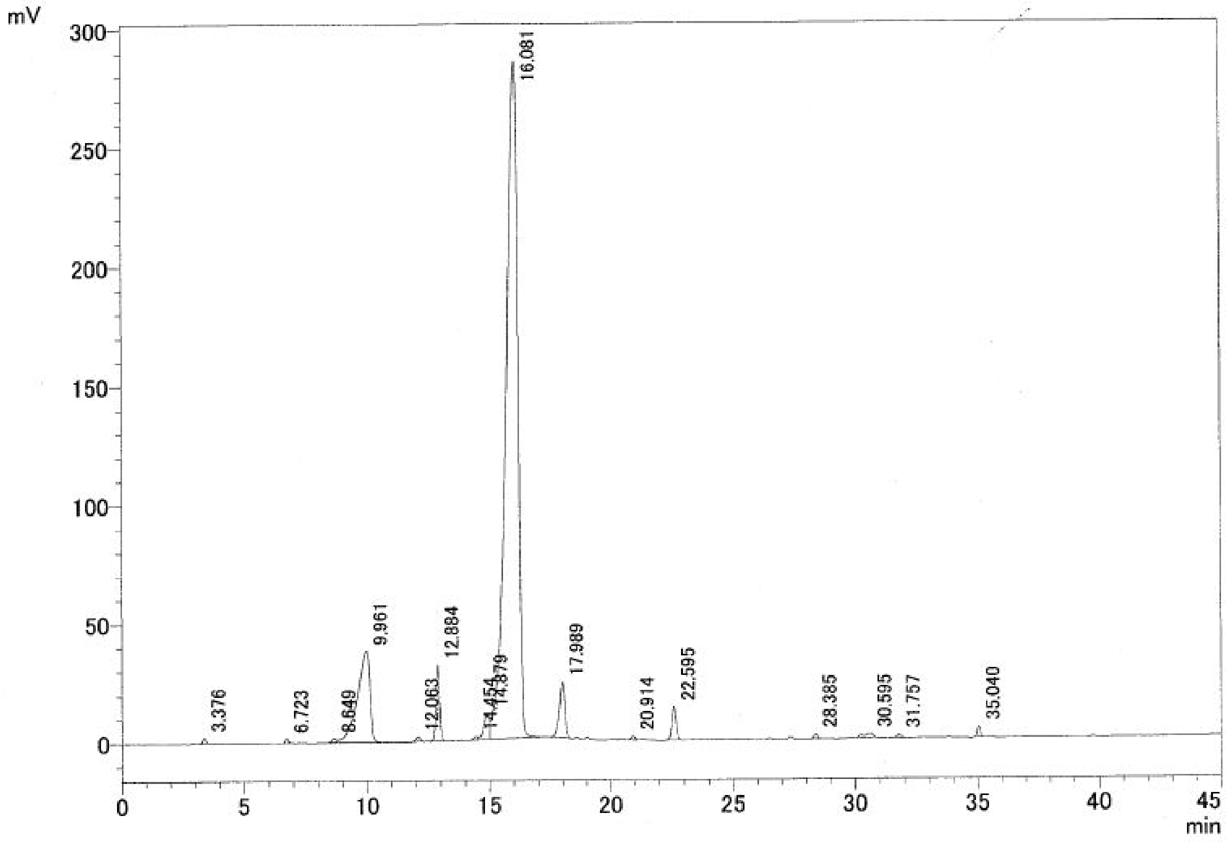

Wave length: 250 nm

Peak #	Compound	Retention time	Area	Height	Area%
1		3.376	17351	2220	0.142
2		6.723	14403	1685	0.118
3	compound 2	8.649	17136	1613	0.140
4	unknown	9.961	1366898	38168	11.161
5		12.063	19807	1719	0.162
6		12.884	326927	31999	2.669
7		14.454	15861	1394	0.130
8		14.879	163816	10983	1.338
9	compound 3	16.081	9645053	284603	78.753
10		17.989	363717	23354	2.970
11		20.914	15416	1474	0.126
12	compound 4	22.595	164266	14043	1.341
13		28.385	20073	2046	0.164
14		30.595	43021	1653	0.351
15		31.757	14629	1419	0.119
16		35.040	38825	4607	0.317
Total			12247199	422981	100.000

Figure S5. HPLC analysis of Napabucasin (**4**) syntheis by flow (Figure 6, reaction solution)

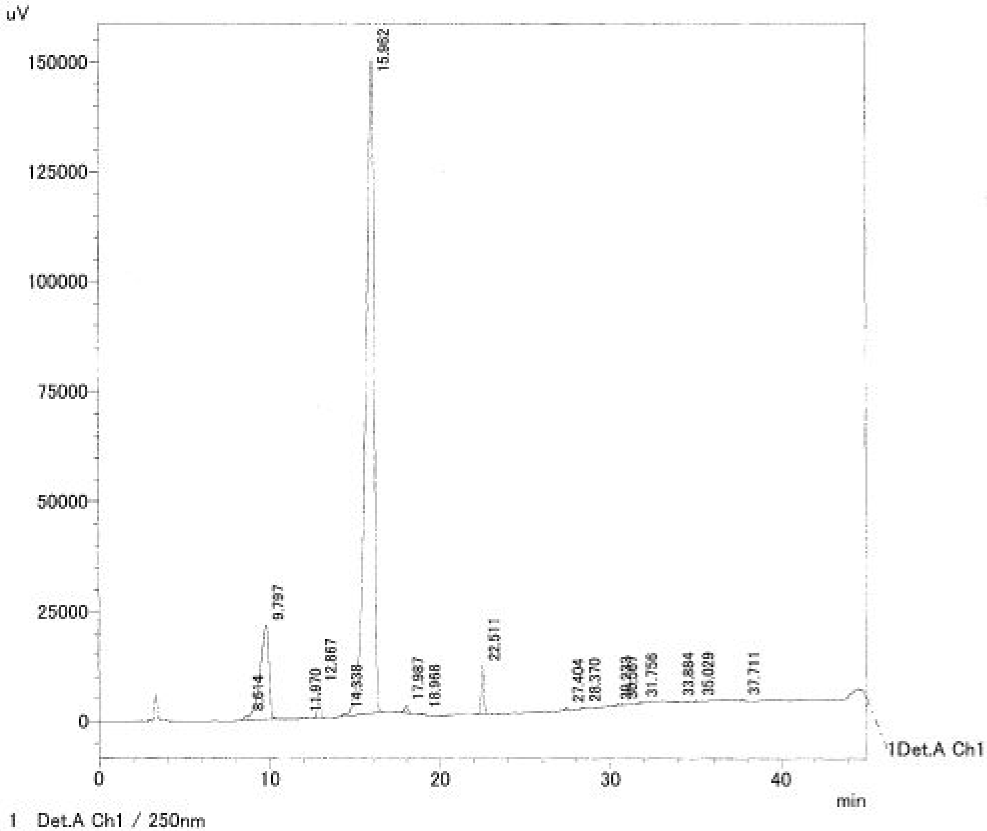

1 Det.A Ch1 / 250nm

Wave length: 250nm

Peak #	Retention time	Area	Height	Area%	
1	8.614	14000	938	0.224	compound 2 (enaminone)
2	9.797	780193	21360	12.486	unknown
3	11.970	3412	252	0.055	
4	12.867	84252	7024	1.348	
5	14.338	9497	640	0.152	
6	15.962	5166643	148569	82.687	compound 3
7	17.987	25793	1664	0.413	
8	18.968	4066	364	0.065	
9	22.511	132498	10918	2.120	compound 4
10	27.404	7146	611	0.114	
11	28.370	4636	436	0.074	
12	30.233	2186	139	0.035	
13	30.567	4867	301	0.078	
14	31.756	2967	264	0.047	
15	33.884	1388	163	0.022	
16	35.029	1438	168	0.023	
17	37.711	3487	182	0.056	
Total		6248468	193995	100.000	

Figure S6. HPLC analysis of Napabucasin (**4**) syntheis by flow (Figure 6, obtained crystal)

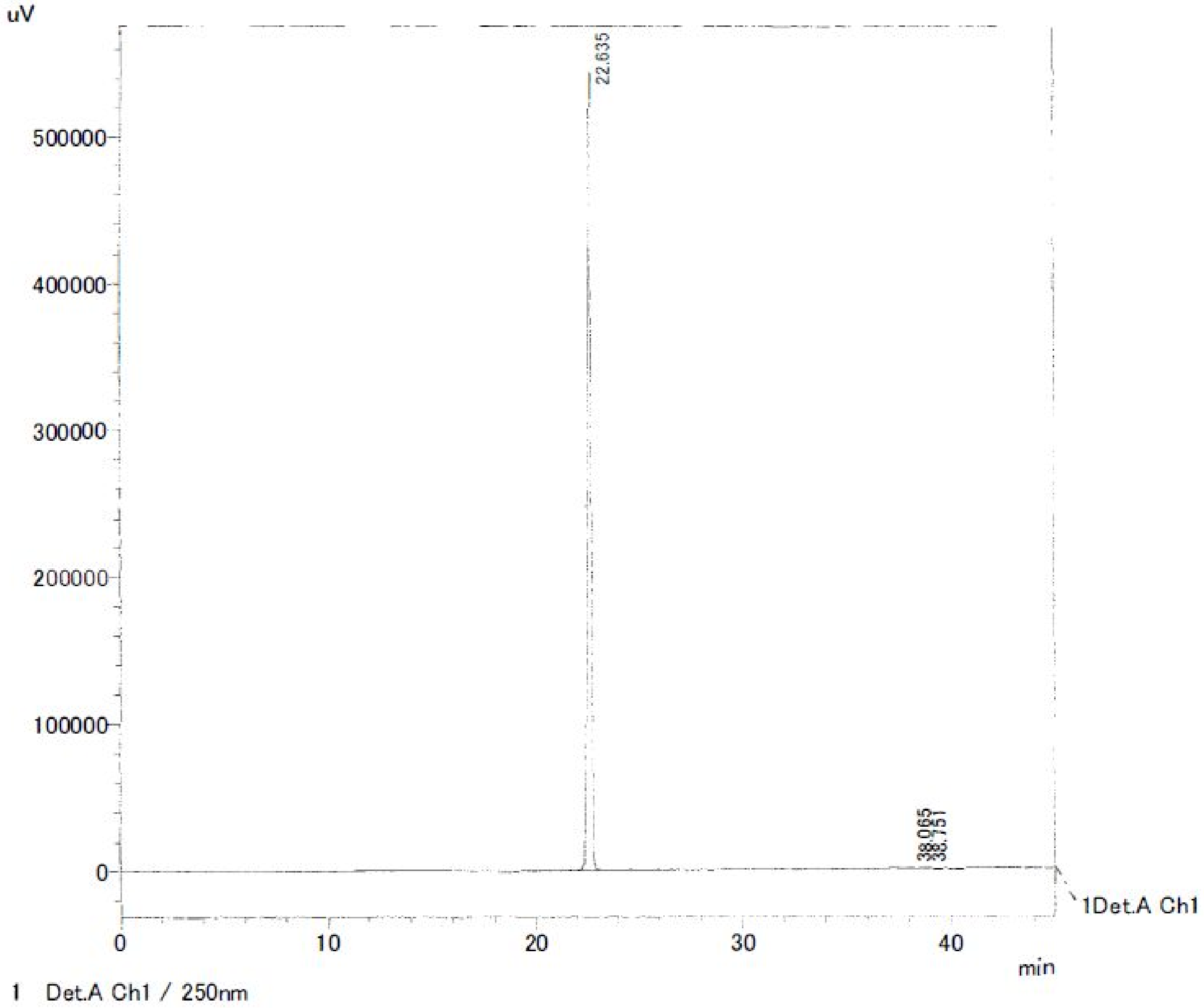

Wave length: 250nm

Peak #	Retention time	Area	Height	Area%
1	22.635	6846452	544407	99.919
2	38.065	3539	365	0.052
3	38.751	2027	254	0.030
Total		6852018	545026	100.000
